# Supplementary material for: Clinical Outcome of Colorectal Cancer Patients with Concomitant Hypertension: A Systematic Review and Meta-Analysis
Source: J Pers Med. 2024 May 14;14(5):520. doi: 10.3390/jpm14050520 (PMC11122181; doi:10.3390/jpm14050520)
Supplement: Supplementary file 1 [file jpm-14-00520-s001.zip › jpm-2954872-supplementary.pdf]

Supplementary file

Table S1. Search string

Database	Search string
Pubmed (Medline)	(((((((("HTN"[Mesh]) OR "Blood Pressure"[Mesh]) OR "Diastole"[Mesh]) OR "Systole"[Mesh]) AND "Colorectal Neoplasms"[Mesh]) OR "Rectal Neoplasms"[Mesh]) AND "Survival"[Mesh]) OR "Mortality"[Mesh]) OR "Disease-Free Survival"[Mesh]).
Embase	1-hypertension<br>2- Blood Pressure<br>3- Diastole<br>4- Systole<br>5- 1 OR 2 OR 3 OR 4<br>6- Colorectal Neoplasms<br>7- Rectal Neoplasms<br>8- 6 OR 7<br>9- Survival<br>10- Mortality<br>11- Disease-Free Survival<br>12- 9 OR 10 OR 11<br>13- 5 AND 8 AND 12
Web of science	1 hypertension<br>2 Blood Pressure<br>3 Diastole<br>4 Systole<br>5 OR/1-4<br>6 Colorectal Neoplasms<br>7 Rectal Neoplasms<br>8 OR/6,7<br>9 Survival<br>10 Mortality<br>11 Disease-Free Survival<br>12 OR/9-11<br>13 5, 8, and 12
Scopus	((hypertension OR Blood Pressure OR Diastole OR Systole) AND (Colorectal Neoplasms OR Rectal Neoplasms) AND (Survival OR Mortality OR Disease-Free Survival))
Cochrane Library	1 Mesh descriptor: (hypertension) explore all trees<br>2 (hypertension*) or (blood pressure*) or (diastole*) or (systole*):ti, ab, kw<br>3 Or 1-2<br>4 Mesh descriptor: (Colorectal Neoplasms) explore all trees<br>5 (Colorectal Neoplasms *) or (Rectal Neoplasms *) : ti, ab, kw<br>6 Or 4-5<br>7 Mesh descriptor: (survival) explore all trees<br>8 (survival *) or (mortality *) or (Disease-Free Survival *): ti, ab, kw<br>9 Or 7-8<br>10 3 and 6, and 9

CINAHL	1 hypertens* OR Blood Pressure [mh] OR diastol* OR systol*<br>2 Colorectal Neoplasms [mh] OR Rectal Neoplasms [mh]<br>3 Survival* OR Mortality* OR Disease-Free Survival [mh]<br>4 1 AND 2 AND 3
